# Molecular and morphological evidences resolve taxonomic ambiguity between *Systomus sarana sarana* (Hamilton, 1822) and *S. sarana subnasutus* (Valenciennes) and suggest elevating them into distinct species

**DOI:** 10.1080/23802359.2018.1481775

**Published:** 2018-08-29

**Authors:** J. R. Biswal, Rajeev K. Singh, Kuldeep K. Lal, Vindhya Mohindra, Rajesh Kumar, Rahul G. Kumar, V. S. Basheer, J. K. Jena

**Affiliations:** a National Bureau of Fish Genetic Resources, Lucknow, India;; b PMFGR Division, National Bureau of Fish Genetic Resources, CMFRI Campus, Kochi, India;; c Indian Council of Agricultural Research, New Delhi, India

**Keywords:** Systomus, molecular, taxonomy, truss, phylogeny, conservation, India

## Abstract

Taxonomic ambiguity exists in genus *Systomus* and recently many new species were described under this genus. *Systomus sarana subnasutus* is considered a valid subspecies of *S. sarana sarana* although revisions have been done by some researchers. We employed a combination of morpho-meristics and molecular tools (*Cytochrome c oxidase I, 16S* and *Cytochrome b* genes of mitochondrial genome) to resolve the two species. Three morpho-meristic characters, head length/maxillary barbel length (HL/MxBL), Lateral Line Scales (LLSs) as well as two truss-based characters, had discernible variation between the two taxa. The sequence analysis (2353 nucleotides) depicted a separate clad of *S. sarana subnasutus* with high bootstrap support. The findings from combined use of morphology, meristics and mitogenes were concordant. The corroborative results suggest the possibility of two different species. The results suggest to adopt suitable management measures, accordingly.

## Introduction

Genus *Systomus* (subfamily: Cyprininae; family: Cyprinidae; order: Cypriniformes) is an economically important group, comprising of 19 fish species native to tropical Asia (Kottelat 2013). *Systomus sarana sarana* (Hamilton 1822) commonly known as olive barb is widely distributed in south-east Asian countries. It has wider occurrence throughout India except peninsular region and south to Krishna River (Talwar and Jhingran 1991). Recent study (Dahanukar 2010) indicated reduction in its natural abundance due to anthropogenic pressures, and places it under vulnerable group. The another subspecies, *S. sarana subnasutus*, popularly known as peninsular olive barb, is endemic to the Western Ghats (Dahanukar et al. 2004) and inhabits the river Krishna and all other rivers south to it (Menon 1963, 1999). Availability of this species in different rivers of peninsular India has also been reported by several other authors (Chandanshive et al. 2007; Jadhav and Yadav 2009; Shahnawaz and Venkateshwarlu 2009).

There have been several revisions for this species. Historically, Valenciennes first described *S. sarana subnasutus* as *Barbus subnasutus* from Pondicherry, India (Cuvier and Valenciennes 1842). Menon (1963) synonymized the species with *Puntius sarana* but considered it as a valid subspecies. Later on, this was considered *Barbodes sarana subnasutus* by Menon (1999). While cataloguing, Eschmeyer and Fricke (2011) again synonymized this species with *P. sarana,* and Jayaram (2010) considered it as a valid subspecies. Dahanukar (2013) had strongly recommended for further taxonomic investigation, especially using molecular markers.

The morphology of fishes has been the key source for taxonomic studies. However, the current trend advocates the potential role of molecular tools to support morphological inferences in resolving ambiguities, especially among the closely related species. Mitochondrial genes are the proven tools to infer variability at inter-/intra-species level, depending upon their evolutionary rates. The slow evolving genes such as COI, 16S have been widely used for inter-species relatedness. In the present study, a combined use of morpho-meristics and three mitogenes was done to resolve taxonomic ambiguity between *S. sarana sarana* and its peninsular congener subspecies, *S. sarana subnasutus*.

## Materials and methods

Fish specimens, used in present study, were collected from commercial catches of various rivers between December 2014 to September 2015 (Table 1). The collected specimens were identified using standard taxonomic keys (Talwar and Jhingran 1991). Digital images were captured for truss morphometry (Karaoglu et al. 2011), at site. For each specimen, a total of 23 traditional morphometric measurements and 10 meristic characters were recorded. A total of 14 landmarks, covering the entire shape of individual specimens, yielded 91 inter-landmarks. The truss distances were extracted using tpsDig2 v2.1 (Rohlf 2006) and PAST (Hammer et al. 2001).

**Table 1.** Specimen information, collection localities and accession number of *Systomus sarana sarana* and *Systomus sarana subnasutus* (*N* is the number of samples analysed).

Sl. No.	Species	Voucher ID	Locality site	Coordinates	NCBI acc. No.
1.	*Systomus sarana sarana*	PSS-241	Lingipur, Odisha, Daya (tributary) Mahanadi River	20^°^14′N 85^°^50′E	NCBI submission ID: 1975367
		PSS-242			
		PSS-245			
		PSS-247			
		PSS-248	Kanasa, Odisha, Luna (tributary) Mahanadi River	20^°^28′N 86^°^22′E	
		PSS-250			
		PSS-251			
		PSS-252	Naraj Barrage, Odisha, River Mahanadi	20^°^28′N 85^°^46′E	
		PSS-254			
		PSS-100	Adilabad, Andhra Pradesh, River Godavari	19^°^02′N 79^°^52′E	
		PSS-101			
		PSS-102			
		PSS-103			
		PSS-104			
		PSS-300	Dhavaleswaram, Rajmundary, Andhra Pradesh, Godavari River	16^°^57′N 81^°^46′E	
		PSS-301			
		PSS-302			
		PSS-303	Bobbarlanka, Rajmundary, Andhra Pradesh, Godavari	15^°^57′N 80^°^52′E	
		PSS-304			
		PSS-266	Ibrahimpatna Vijayawada, Andhra Pradesh, Krishna River	16^°^31′N 80^°^36′E	
		PSS-267			
		PSS-268			
		PSS-269			
		PSS-270			
		PSS-271			
		PSS-272			
		PSS-273			
		PSS-274			
		PSS-276			
		PSS-275			
		PSS-277			
2.	*Systomussubnasutus*	PSN-6	Thakaji, Kerala, Manimala River	09^°^22′N 76^°^24′E	NCBI submission ID: 1975582
		PSN-7			
		PSN-8			
		PSN-9			
		PSN-10			
		PSN-11			
		PSN-12			
		PSN-13			
		PSN-14			
		PSN-15			
		PSN-1	Aluva , Kerala Periyar river	10^°^06′N 76^°^20′E	
		PSN-2			
		PSN-3			
		PSN-4			
		PSN-5			
		PSN-16			
		PSN-17			
		PSN-18			
		PSN-19			
		PSN-20			
		PSN-21			
		PSN-22			
		PSN-23			
		PSN-24			
		PSN-25			
		PSN-26			

### Genetic analysis

#### Gene sequencing

Total gDNA was extracted from individual muscle tissue samples using the phenol–chloroform method. The protocol of Ruzzante et al. (1996) with slight modification (Singh et al. 2012) was followed. The amplification reaction was set in ABI Veriti thermocycler. The universal primers were used for amplifying Cyt b (Xiao et al. 2001); 16S rRNA (Palumbi et al. 1991) and COI (Ward et al. 2005). The amplicons were sequenced bi-directionally on ABI 3730 sequencer.

### Data analysis

#### Statistical analysis

To determine inter-specific variations, traditional morphometric and meristic characters were used separately in analysis as their allocation abilities are different, statistically (Karaoglu and Belduz 2011). Species were compared using ratios among various traditional morphometric characters and mean values of meristic characters. A one-way analysis of variance (ANOVA) was conducted on each of the variables for principal component analysis (PCA) and discriminant function analyses (DFA).

The raw DNA sequences were edited and aligned using BioEdit software version 7.0.5.2 (Hall 1999) and Clustal W (Thompson et al. 1997). A total of 655 bp (COI), 557 (16S) and 1141 (Cyt b) were analysed. The sequence characteristics, such as polymorphic sites, nucleotide composition, and transition/transversion ratios were determined.

To ascertain the inter-relatedness of the two subspecies of *S. sarana*, gene sequences of *S. orphoides*, *S. chalakkudiensis* and *S. denisonii* were downloaded from NCBI GenBank and analysed together. Neighbour joining (NJ) tree was generated with 1000 replications (Saitou and Nei 1987).

## Results

### Morpho-meristics and Truss network

The ANOVA revealed eight morphometric ratios and four meristic characters to exhibit significant (*p* < .05) differences between the two taxa. However, discriminant analysis of significant variables demonstrated three variables namely, head length/maxillary barbel length (HL/MxBL), lateral line scales (LLSs) and vertebrae counts (VCs). Significant (*p* < .05) Fisher’s distances between the groups were observed.

The ANOVA revealed that the samples of two species differed significantly (*p* < .05) at 57 transformed morphometric characters, out of a total 90. PCA extracted 10 principal components accounting for 92.2% of the total variation. HL/MxBL, LLSs, VCs, eye diameter and distance between pectoral fin origin to operculum differentiated the two *Systomus* species.

### Molecular analysis

The multiple alignment of COI gene (655 bp) from two species, *S. sarana sarana* (19) and *S. sarana subnasutus* (18), revealed six haplotypes, with two and four haplotypes were found, respectively (Table 2). A total of 636 sites (97.1%) were conserved 19 (2.9%) variable and 17 (2.6%) parsimony informative. The analysis depicted the average nucleotide frequencies as A = 27.9%, T = 28.4%, G = 17.2% and C= 26.5% which showed higher AT (56.3%) than GC content (43.7%). The order of occurrence of bases was T > A > C > G in both the species. Overall, the occurrence of transitional events was more commonly observed than transversions.

**Table 2.** Haplotypes and variable sites of COI sequences.

Nucleotide position →										1	1	1	1	1	1	1	1	1	1
	1	2	3	4	5	6	7	8	9	0	1	2	3	4	5	6	7	8	9
S species↓	T	G	C	G	G	A	A	G	G	G	G	G	C	A	T	T	C	A	T
*S. sarana* - H1	C	A	T	A	.	G	G	A	A	.	A	A	T	G	C	G	.	.	C
*S. sarana* - H2	C	A	T	A	.	G	G	A	A	.	A	A	T	G	C	G	.	G	C
*S. subnasutus -* H1	.	.	.	.	.	.	.	.	.	.	.	.	.	.	.	.	.	.	.
*S. subnasutus* - H2	.	.	.	.	.	.	.	.	.	A	.	.	.	.	.	.	.	.	.
*S. subnasutus* - H3	.	.	.	.	T	.	.	.	.	A	.	.	.	.	.	.	.	.	.
*S. subnasutus* - H4	.	.	.	.	T	.	.	.	.	A	.	.	.	.	.	.	T	.	.

**Figure 1.**
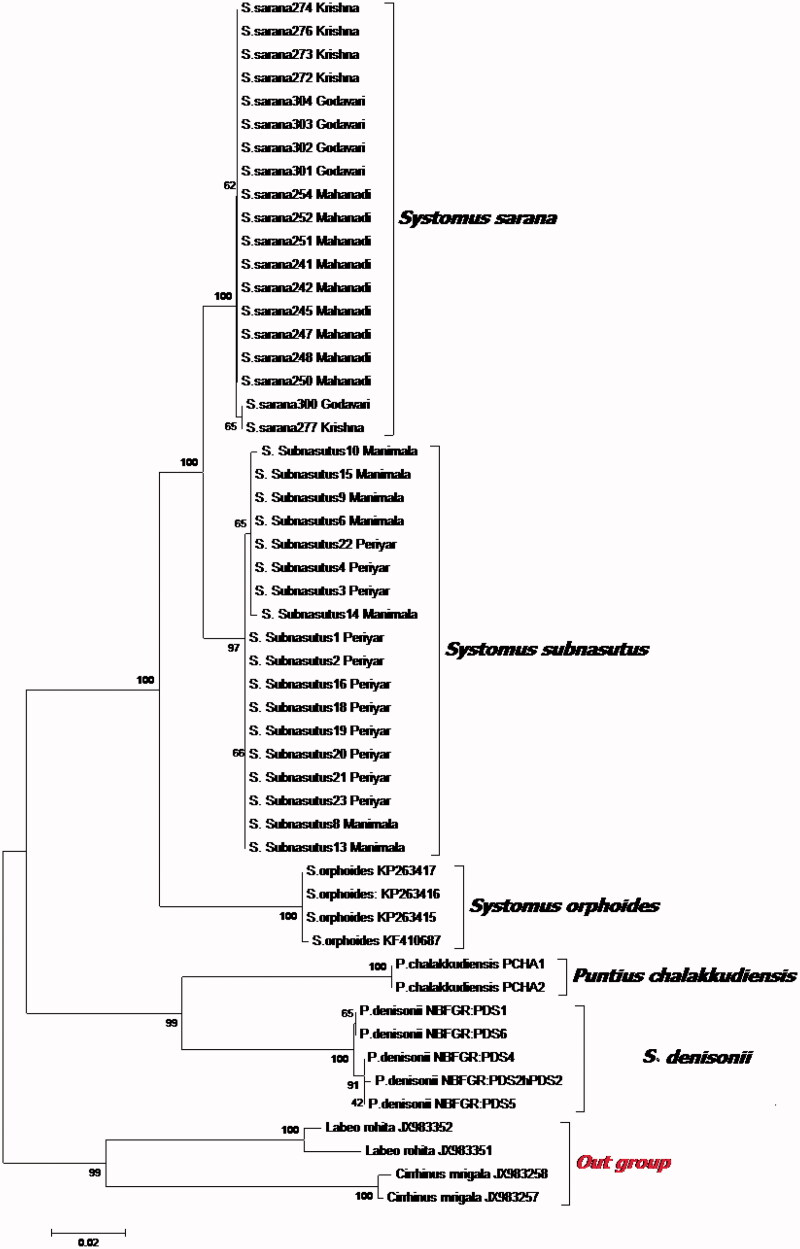
Phylogenetic relationships of *Systomus* species with close relatives based on K2P divergence of COI gene.

The genetic distance within populations (for both taxa) did not exhibit significant differences. However, the average genetic distance between the *S. sarana subnasutus* and *S. sarana sarana* were 2.4% and 2.5%, respectively (Table 3). NJ tree was constructed (K2P distance) using all 37 COI sequences including congeners from NCBI. Monophyly of *Systomus* (*Sahayadria*) group was presented when Indian major carp species were used as outgroups. However, the polyphyletic clustering was obvious for *Systomus* and *Sahayadria* genera. The topology is similar to our own work (under review) which supported the distinctiveness of three taxa (Figure 1). Furthermore, strong bootstrap support indicated the divergence of *Systomus* genera with that of *Sahayadria* which is in line with conventional taxonomy. The tree shows 100% bootstrap to support distinction between two taxa, *S. sarana sarana* and *S. sarana subnasutus*.

**Table 3.** Genetic distance between *S. sarana subnasutus* and *S. sarana sarana*.

Species	*S. sarana subnasutus* (Periyar)	*S. sarana subnasutus* (Manimala)	*S. sarana sarana* (Mahanadi)	*S. sarana sarana* (Godavari)	*S. sarana sarana* (Krishna)
*S. sarana subnasutus*, (Periyar)	0.00	0.001	0.006	0.006	0.006
*S. sarana subnasutus,* (Manimala)	0.001	0.00	0.006	0.006	0.006
*S. sarana sarana,* (Mahanadi)	0.024	0.025	0.00	0.000	0.000
*S. sarana sarana,* (Godavari)	0.024	0.025	0.000	0.00	0.000
*S. sarana sarana* (Krishna)	0.024	0.025	0.000	0.000	0.00

Below diagonal are the Fst values, above diagonal are the significance level.

**Figure 2.**
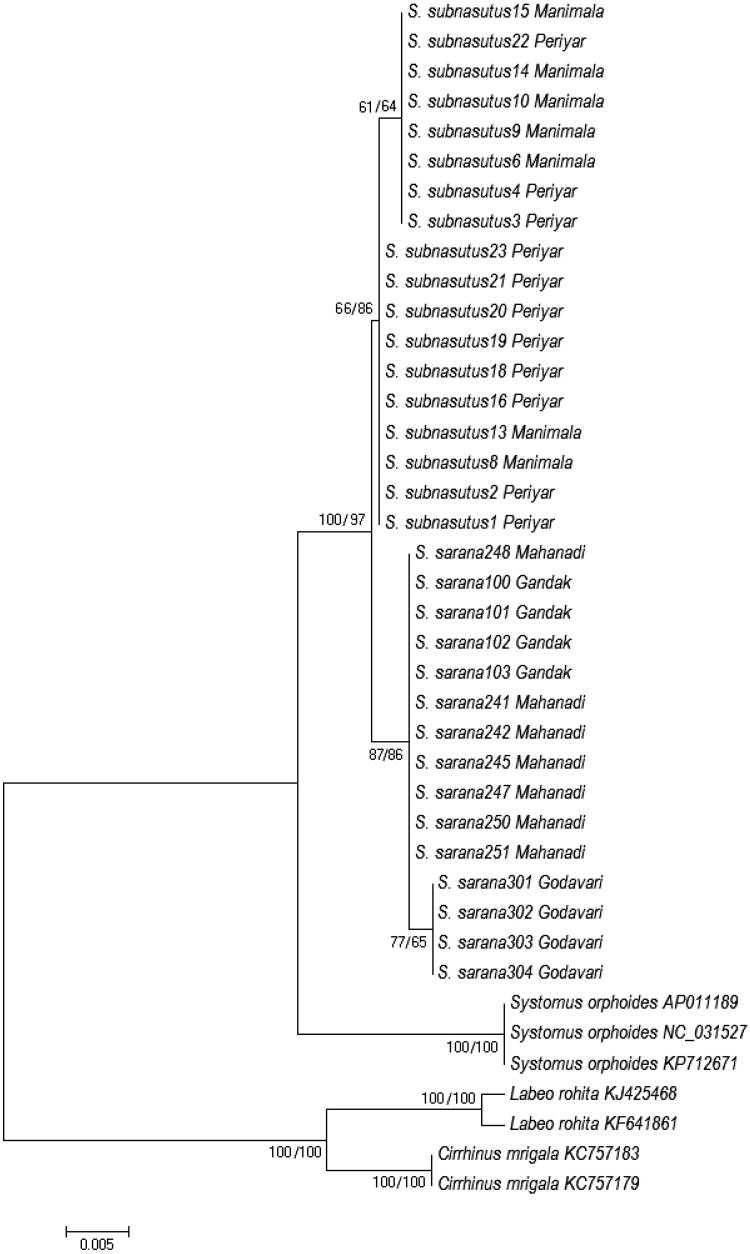
Phylogenetic relationships of *Systomus* species with close relatives based on two mitogenes. Bootstrap values on the nodes are for gene Cyt (before) and 16S (after) slash.

Sequence analysis of 16S rRNA (557 bp) revealed two haplotypes, each from *S. sarana sarana* (*n* = 15) and *S. sarana subnasutus* (*n* = 18). The genetic distance was 0.18% within taxa, while 0.5% observed between individuals of *S. sarana sarana* and *S. sarana subnasutus.* Analysis of Cytochrome b (1141 bp) presented 1090 conserved sites, whereas 51 were variable ones. Pairwise genetic distance between *S. sarana sarana* and *S. sarana subnasutus* was 3.73%, whereas different populations of both the species showed low genetic distance (0.89% and 0.35%), respectively. The NJ tree highlighted similar tree topologies with high bootstrap (71–100) (Figure 2) for both mtDNA markers.

## Discussion

Previously, several researchers demonstrated the taxonomic status of both species (Menon 1999; Jayaram 2010; Eschmeyer and Fricke 2011). The genus *Puntius* has been considered one of the largest group among subfamily cyprininae. This subfamily is represented by over 120 valid species, widely distributed in south and south-east Asia. With all these revisions, the species of genus *Puntius* (Hamilton 1822) along with *P. sophore* as type species are recognized under the six distinct genera *Puntius, Systomus, Dawkinisia, Haludaria* (*Dravidia*), *Sahyadria* and *Pethia*. Sarana group of genus *Systomus* is a complex consisting of *S. sarana sarana* and its three valid subspecies viz. *S. sarana spilurus*, *S. sarana subnasutus* and *S. sarana orphoides.*

Traditional morphology is often used for species identification by gathering and analysing, data from large sample sizes, though molecular markers are proven to be more robust in reconstructing phylogenies. The combined use of the morphological characters and mitochondrial data provides a strong framework for discriminating species, compared to the use of morphological characters alone. In the present investigation, we used molecular evidences (along with morphological attributes) to corroborate the taxonomic status of two species.

The overall results from this study indicated that *S. sarana sarana* differs significantly from *S. sarana subnasutus*. Earlier, Jayaram (1991), in his study on genus *Puntius* reported that in *P. sarana sarana* (Hamilton), LLSs are 30–34 with mostly 31 or 32; and all populations are distributed north of the Krishna river system in Southern India, while its subspecies, *P. sarana subnasutus* (Val.), the LLSs range 28–31 and all populations were distributed in Krishna river (and south to it) in the Peninsular India. In *P. sarana sarana* contains a dark blotch on lateral line before base of caudal fin which is distinguishable with the similar blotch at 24th scale in *P. sarana subnasutus* (Jayaram 2010).

In *S. sarana sarana,* eye diameter is larger than *S. sarana subnasutus*. Similarly, distance between pectoral fin origin to operculum is larger in *S. sarana sarana* compared to *S. sarana subnasutus*. In this study, truss morphometry also yielded concordant results. Truss-based differentiation has been used in several fish species (Cavalcanti et al. 1999; Parsons et al. 2003; Sakai et al. 2009). The analysis of 2353 nucleotides, further confirmed the possibility of differentiation between *S. sarana sarana* and *S. sarana subnasutus* on the basis of NJ phylogram which was supported by high bootstrap values in all three mitochondrial markers. The pattern of clustering demonstrated their separate existence.

Conclusively, with the molecular evidence corroborating to the morphological inferences, this study strongly advocates that there is probability that *S. sarana subnasutus* is a valid species and distinct from the *S. sarana sarana*. However, taxonomic re-description is required for elevating from subspecies to species level. The findings are important for conservation and management of resources.
